# Early free light chain reduction following treatment initiation predicts favorable outcome in intact immunoglobulin myeloma

**DOI:** 10.1038/s41408-021-00600-6

**Published:** 2022-01-05

**Authors:** Jean-Sébastien Claveau, Sophie Savary Bélanger, Imran Ahmad, Jean-Sébastien Delisle, Vincent De Guire, Jean Roy, Richard LeBlanc

**Affiliations:** 1 Division of Hematology, Oncology and Transplantation, Department of Medicine, Maisonneuve-Rosemont Hospital, Université de Montréal, Montreal, QC Canada; 2 Department of Biochemistry, Maisonneuve-Rosemont Hospital, Université de Montréal, Montreal, QC Canada

**Keywords:** Myeloma, Preclinical research

**Dear Editor,**

Multiple myeloma (MM) is characterized by the proliferation of malignant plasma cells [1]. The neoplastic clones usually secrete an intact immunoglobulin (iIg) or fragments of immunoglobulin (Ig) such as light chains. Intact immunoglobulins are traditionally measured by serum (SPEP) and urine protein electrophoresis, which are currently the gold standards to assess MM [2]. However, due to long half-lives of iIgs, SPEP is limited in its capacity to detect early changes after initiation of treatment.

MM displays enormous clonal heterogeneity with myeloma cell clones secreting iIg while other clones secreting the corresponding free light chain (FLC). These serum FLCs (sFLCs) have a short half-life of a few hours depending on renal function. The role of sFLCs as a prognostic factor at baseline and in the assessment of stringent complete response (sCR) is well established [3, 4]. In addition, Ig heavy/light chain analysis (HLC) combines measurement of individual Ig heavy chain isotypes with either κ or λ light chains [5]. The analysis allows accurate quantification of the involved and uninvolved Igs and permits to obtain a ratio of monoclonal and polyclonal Igs of the patient’s affected isotype.

The primary aim of this study was to investigate the clinical value of serial sFLC and HLC measurements during the early phase of treatment, compare them to standard serum M-protein quantification by SPEP and evaluate the impact on progression-free survival (PFS).

Between 2012 and 2014, consecutive newly diagnosed and relapsed MM patients with measurable disease by iIg (≥10 g/L) and sFLC (≥100 mg/L) were prospectively identified [6]. Patients on dialysis were excluded. Informed consent was obtained in all patients in accordance with the Declaration of Helsinki

Serum samples for quantitative measurement of the paraprotein were obtained at initiation of treatment and thereafter every week for the first 3 cycles. The duration of each cycle was 3 or 4 weeks depending on the treatment regimens. SPEP was performed on Hydrasis (Sebia,) and sFLC with the Freelite^®^ assay (The Binding Site Inc) on an Immage nephelometer (Beckman Coulter). HLCs were measured using the Hevylite® assay (The binding Site Inc) on the BN II (Siemens Healthineers).

Responses for each paraprotein assay were descriptive and based on % of reduction compared to baseline: ≥90% reduction versus ≥50% reduction versus <50% reduction. The unstable response was defined as an unsustained decrease in sFLC by ≥25% followed by an increase by ≥25% and ≥25 mg/L within the same cycle. A 90% cutoff for involved sFLC was used to make easier the comparison with the 90% cutoff for serum M-protein as per IMWG uniform response criteria [6].

Multivariate logistic regression was used to assess the association between best paraprotein response by SPEP and achieving ≥50% of paraprotein reduction after cycle 1 by sFLC. The probabilities of PFS were estimated using the Kaplan-Meier method. The designated time point for landmark analysis was the end of cycle 1 and the end of cycle 3. Regression analysis using Cox proportional hazard regression models was used to determine factors influencing outcomes. Statistical analyses were conducted using SAS (SAS Institute Inc).

We evaluated 30 episodes of treatment among 24 patients. The median age was 63 years (range: 39−89). Per eligibility criteria, all MM episodes studied secreted an iIg: 23 IgG and 7 IgA. Treatment regimens were bortezomib-based in 57%, lenalidomide-based in 23% or pomalidomide-based in 17% of patients. Seven episodes of treatment were in newly diagnosed MM patients, the remaining episodes in relapsed disease (median of 4 prior lines of treatment; range 2−8). Patients received an autologous stem cell transplant in 12 episodes of treatment and an allogeneic transplant in one. In addition, 11 episodes (37%) were in patients participating in clinical trials receiving experimental agents in combination with a bortezomib- or pomalidomide-based treatment. The median follow-up was 85 months. By the end of cycle 1, a ≥50% reduction was achieved in 20% by SPEP, 53% by sFLC, and 27% by HLC. This reduction by sFLC preceded the one by SPEP by a median of 3 weeks and the one by HLC by a median of 2.5 weeks.

In addition, 7 of 11 (64%) treatment episodes who did not achieve ≥50% paraprotein reduction by SPEP did not achieve ≥50% of reduction by FLC. Response by sFLC at the end of cycle 1 correlated with the best response achieved by SPEP on treatment, while these observations were not seen with HLCs.

**Fig. 1:**
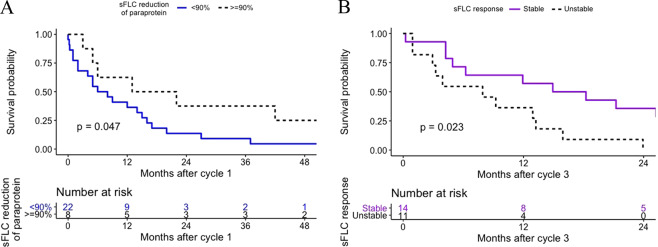
Progression-free survival according to sFLC response. **A** Shown is the progression-free survival (PFS) according to sFLC reduction of paraprotein after cycle 1 and **B** progression-free survival (PFS) by the stability of early sFLC response.

Patients with paraprotein reduction measured by sFLC ≥90% by the end of cycle 1 had a better 1-year PFS, compared to those with <90% reduction (63% vs. 36%, *p* = 0.047; Fig. 1A). After multivariate adjustment for ISS (HR 1.09, *p* = 0.883), reduction of paraprotein ≥50% by SPEP after cycle 1 (HR = 1.79, *p* = 0.427), only reduction of paraprotein ≥90% by sFLC after cycle 1 (HR 0.23, *p* = 0.011) remained predictive of better PFS.

Similarly, among patients with <50% paraprotein reduction by SPEP at the end of cycle 1, those having a ≥90% paraprotein reduction by sFLC showed a longer 1-year PFS compared to those with less than 90% reduction (100% vs. 35%, *p* = 0.049). In the multivariate logistic regression, achieving ≥50% of paraprotein reduction by sFLC after cycle 1 was strongly associated with a late reduction of ≥50% with SPEP during the study (OR 24.09, *p* = 0.004), but not with ISS (OR = 1.31, *p* = 0.813). The sensitivity and specificity of our prediction model were respectively 81 and 89%.

Using sFLCs, patients with unstable response had a significantly worse 1-year PFS compared to patients with stable response (36% vs. 57%, *p* = 0.023, Fig. 1B) regardless of iIg results at end of cycle 3. In multivariate analysis, ISS was stratified, and only unstable response was predictive of progression (HR 3.42, *p* = 0.037). SPEP and HLC paraprotein fluctuations were not observed.

This study is the first to prospectively study and compare early treatment responses by SPEP, sFLC, and HLC on the outcome of iIg MM. sFLCs are a good real time indicator of tumor burden, including in iIg MM. As reported by Mead [7] and Pratt [8], we confirm that the decrease in paraprotein measured by SPEP and sFLCs occurred early after treatment initiation, with the latter showing a more rapid reduction due to a shorter serum half-life of sFLCs. We also confirmed previous observations made by Dispenzieri et al. who reported that sFLCs allow detection of early response among iIg MM and correlate with a favorable prognosis [9]. In our study, we evaluated patients receiving modern treatments, including bortezomib and IMiDs with several paraprotein evaluations. Our data indicate that early assessment of sFLCs as early as cycle 1 has a significant impact on prognosis. Our results are also consistent with Holzhey et al. who demonstrated that rapid sFLC decrease on day 8 is an early prognostic marker for newly diagnosed patients undergoing bortezomib-based treatments [10].

Hansen et al. demonstrated that a > 80% reduction in sFLCs 21 days after initiation of treatment was strongly associated with the achievement of ≥VGPR [11]. Their results are consistent with the fact that obtaining a normal sFLC ratio confers a more favorable prognosis, irrespective of the depth of response [12]. Similarly, we have shown that patients failing to reduce sFLCs by at least 50% by the end of cycle 1 had significantly shorter PFS, independently of ISS and type of treatment received.

Few studies have reported the outcome of patients experiencing unstable sFLCs [11, 13, 14]. These fluctuations likely reflect a temporary inhibition of tumor synthesis rather than tumor killing [13] and could be an early indication of tumor resistance, not immediately evident in serial measurements of SPEP due to a longer half-life of iIgs. In our study, patients with an unstable response using sFLC assay had a significantly worse prognosis.

In contrast to sFLCs, there is little published data on the use of HLC monitoring after initiation of treatment. The response was detected at the same time by HLC and SPEP in most of our patients [15], which is unsurprising considering the serum half-life of heavy chains. Response by sFLC was evident much earlier. Of interest, Bradwell et al. [5] reported a case of early progression in a patient with IgG MM and increasing HLC ratio during the first chemotherapy cycle while the M-protein was decreasing. A rapid paraprotein increase was observed after cycle 2 with both assays. One of our patients behaved similarly, concomitantly with an unstable sFLC levels.

This single-institution, prospective study has limitations, including the fact that a small sample size limits detection of differences in PFS between each different groups with a heterogeneous population. Lack of bone marrow aspirate/biopsy and urinary Bence Jones evaluations also limited correlations with IMWG response criteria. Nonetheless, our results show that early reduction in sFLC is clearly predictive of a better clinical outcome and the assay could potentially be used to optimize therapy.

In conclusion, patients with iIg MM presenting early sFLC improvement at the end of cycle 1 have a better prognosis, even without apparent response by SPEP. Moreover, patients with unstable sFLC response have significantly shorter PFS. Although our observations need to be confirmed in larger cohorts of patients, we believe that early sFLC measurements are predictive of clinical outcomes and could be used to personalize therapy.
